# Illuminating the nutrition-related policy-practice gaps in colorectal cancer survivorship

**DOI:** 10.1007/s00520-024-08332-6

**Published:** 2024-01-25

**Authors:** Kristina Vingrys, Lauren Atkins, Eva Pape, Annelie Shaw, Amanda Drury

**Affiliations:** 1 Institute for Health and Sport, Victoria University, PO Box 14428, Melbourne, VIC 8001 Australia; 2 First Year College®, Victoria University, PO Box 14428, Melbourne, VIC 8001 Australia; 3 OnCore Nutrition, 863 Glen Huntly Rd, Melbourne, VIC 3162 Australia; 4 Cancer Center, Ghent University Hospital, Corneel Heymanslaan 10, Ghent, Belgium; 5 School of Public Health, Physiotherapy and Sports Science, University College Dublin, Dublin, Ireland; 6 School of Nursing, Psychotherapy and Community Health, Dublin City University, Glasnevin, Dublin 9, Ireland

**Keywords:** Colorectal cancer, Survivorship, Quality of life, Unmet needs, Nutrition care, Policy-practice gaps

## Abstract

**Purpose:**

Colorectal cancer (CRC) is among the three most commonly diagnosed cancers globally, after breast and lung cancer, with an estimated 2 million new cases each year, comprising ten per cent of all cancers worldwide. CRC has a complex aetiology associated with several nutrition-related risk factors. Cancer survivors frequently report alterations to their dietary habits and nutritional intake, with related adverse impacts on health-related quality of life (QOL). Whilst nutrition-related factors are recognised as survivor priorities and embedded in survivor care policies, dietary support is frequently not the standard of care in practice.

**Methods and results:**

In this Commentary, we present details of a critical policy-practice gap for CRC survivors across the spectrum of nutrition care that we have seen growing in the literature, in hospitals, community and private practice.

**Conclusion:**

As these nutrition concerns can adversely impact QOL and morbidity and mortality risks, we hope to raise awareness of these issues to provide a basis of future work in this area, so that policymakers and clinicians can improve support and outcomes for CRC survivors and their families.

## Background

CRC survivors face unique nutrition-related challenges in self-managing their health following cancer surgery, radiotherapy, chemotherapy and biological therapies, encompassing bowel management, ostomy care and diet and lifestyle changes, each requiring significant practical and psychological support to facilitate adjustment [1]. Moreover, different challenges between colon and rectal cancer survivors may be apparent, including low anterior resection syndrome (LARS) in rectal cancer survivors with risks of long-term appetite reduction, diarrhoea, incontinence, rectal bleeding, pain and obstruction associated with pelvic exenteration and radiotherapy [2]. People who require ostomies following colon and/or rectal cancer report difficulties from the ostomy, including increased anxiety and reduced QOL relating to managing equipment malfunction and bowel control, with reports including risks of leakage and accidents, constipation, obstruction, diarrhoea and urgency, incontinence, flatulence and discomfort [2, 3]. The chronic bowel dysfunction and related deteriorations in health-related QOL experienced by both colon and rectal cancer survivors are lacking evidence-based interventions; however, diet modification via behaviour change interventions may be useful targets [2].

## Nutrition-related factors in CRC survivorship

Nutrition interventions can positively impact CRC survivor outcomes including nutritional status, dietary intake and QOL [4]; however, diet and lifestyle information is frequently lacking among people living with and after CRC (Table 1), particularly in the transition period post-treatment [1, 3]. Changes in diet have as much to do with self-management and QOL goals, including managing challenges associated with bowel or ostomy function (Table 1), as to control the trajectory of disease and prevent recurrence or new primary cancers [1, 3]. Bowel control is a significant challenge for CRC survivors that may incorporate a systematic regimen of medication coupled with evidence-based dietary counselling, dietary intervention and psychological support [5]. In actual practice, bowel management strategies appear to be self-managed by survivors using a trial-and-error approach [1, 3], often due to lack of professional guidance available (Table 1) [1, 3].

**Table 1:** Synthesis of nutrition-related effects and aetiological foundations related to unmet nutrition-related needs reported by CRC survivors

Nutrition-related effects and aetiology	References
Support adjusting to living with a colostomy bag and effects post-surgery *Aetiology:* Lack of guidance, information and support, feeling ‘ill-equipped’ to manage this major life change; Lack of understanding or empathy from healthcare workers; Lack of availability of ostomy nurses; Balancing vigilant surveillance, self-awareness and fear of ‘crying wolf’ *Nutrition-related effects*: Difficulty adjusting to living with a colostomy; feelings of distress; financial burden paying for private services to mitigate potential health service delays; loneliness, fear and anxiety	[3, 6]
Appropriate nutrition advice *Aetiology*: Conflicting and counterintuitive messages about what to eat; Unaware of the links between CRC incidence and recurrence, diet and lifestyle; Being provided with ‘broad’ and ‘useless’ information; Not receiving enough dietary information and lack of counselling; Medical consultations referring to patients’ ‘unhealthy diets’ when discussing possible causes of CRC, without context of actual dietary practices *Nutrition-related effects*: Adopting a ‘trial and error’ approach, necessitating dietary modification in response to problems with bodily function; feelings of frustration; unsupported causal beliefs about diet lifestyle factors to reduce disease risk in future; overly restrictive diets and nutritional inadequacies, reduced confidence; unable to achieve health and weight goals; food-related fear, guilt and shame, feeling stigmatised	[1, 3, 7]

Where formal interventions are in place to support the management of bowel dysfunction in the post-operative period, a scoping review of 10 papers and 12 intervention pathways for LARS following sphincter-saving surgery found interventions are poorly described with limited fidelity to the planned treatment, lacking a single definitive intervention pathway, unclear rationale for use within most multi-modal pathways and intervention variations including number and type of interventions, intervention timing and patient eligibility for intervention [8]. Furthermore, due to the limited evidence, only some of these reported interventions utilised dietary interventions, despite this being a frequently reported self-management strategy for bowel dysfunction among CRC survivors [8]. CRC survivors have employed numerous self-managed dietary strategies [2]; however, worse health-related QOL is associated with long-term survivors who report difficulties in making dietary adjustments [2] representing an opportunity for early intervention to improve survivor well-being [2].

## Dietary intervention and potential barriers

Dietary consultation is rarely a standard of care for people receiving treatment and follow-up for CRC [7, 9] despite a consensus of evidence and guidelines recommending cancer survivors receive nutritional care advice and education from trained professionals [10–13]. Data from the Victorian Cancer Malnutrition Collaborative 2022 Point Prevalence Study found more than 50% of CRC patients received no nutrition support, only 27% of CRC patients received dietetic intervention, and less than 50% of CRC patients who were identified as malnourished had received nutrition intervention from a dietitian [9]. CRC patients were also in the top five tumour types for the largest shortfalls in dietetic services to malnourished cancer patients by volume of ~ 6%, and along with breast cancer, CRC represents one of the top five cancer streams with the largest shortfalls in dietetic intervention to patients with malnutrition in each of the Victorian cancer malnutrition point prevalence studies (2012, 2014, 2016, 2018, 2022) [9]. Lack of dietetic outpatient follow-up for CRC patients post-surgery is also reported in other studies [7].

Nutrition care and dietary intake in cancer are recognised as fundamental to recovery and survival (WCRF & AICR, 20189), and nutritional guidance remains a widely reported practice gap in cancer care generally [9] and in CRC [2, 7, 9]. Policy-practice gaps include a lack of access to CRC dietitians, medical nutrition support and nutrition information (Table 1) [7]. Multi-disciplinary care teams, including dietitians, provide specialised nutrition intervention and counselling to cancer patients, and families, stomal therapy nurses and medical practitioners are also called upon, as specialised nutrition practitioners are under-utilised and under-resourced to provide nutrition care [7]. Barriers may include low nutrition screening rates and dietitian referrals, insufficient medical and primary care awareness of patient needs and available nutrition and supportive care services, geographical and financial access constraints, and availability of resources in treating facilities and the community [14].

## Position statements and guidelines

Whilst a range of nutrition-related policy-practice gaps in CRC survivorship are emerging in the literature, there seems to be a concomitant gap in position statements and management guidelines to address the nutrition-related needs in the CRC survivorship spectrum. The nutritional impacts of CRC (and cancers generally) include sarcopenia and malnutrition during treatment [12]; malnutrition and sarcopenia have implications for outcomes after a cancer diagnosis, including mortality and QOL [15]. Whilst these seem to be better addressed, possibly due to resource allocation to improve malnutrition and sarcopenia management during treatment, intervention is still insufficient. Prado and colleagues’ recent position paper [13] presents a comprehensive summary from fourteen expert organisation recommendations in oncology nutrition to improve patient care [13]. These include the European Society for Clinical Nutrition and Metabolism (ESPEN) expert group recommendations for action against cancer-related malnutrition [11], ESPEN practical guideline: Clinical Nutrition in cancer [16] and Clinical Oncology Society of Australia (COSA) position statement on cancer-related malnutrition and sarcopenia [17]. These recommendations recognise the importance of a multi-modal approach [11, 13, 16], multi-disciplinary teams [13, 17] and individualised counselling [11, 16, 17]; however, their focus is primarily on malnutrition and sarcopenia, medical priorities that may be under-treated in cancer generally [11, 17] but not specifically aligned or intended to address the unmet nutrition-related needs of CRC survivors. Management guidelines for LARS [18] and colorectal cancer survivorship [19] recognise the role of dietetic advice; however, it is noted in practice that patient trial-and-error is applied in diet manipulation and symptom management [1, 3]. Moreover, a recent European consensus highlights the fundamental nature of integrating nutrition care for all cancer patients to receive patient-centred medical care, as not only a human right, but as clinicians’ ethical duty, and reminding us of the core patient care principle of “primum non nocere” [20].

## Conclusion

Evidence is mounting that a policy-practice gap in CRC survivor nutrition-related factors and services exists across the survivor experience and is impacting health-related QOL, morbidity and mortality. We suggest it is time to address these important issues and are calling for action to understand how medical and allied health professionals can better support CRC survivors and their families. We suggest a useful starting point is a comprehensive review to inform future consensus guidelines, policy implementation and funding commitment, from local to global health and governmental levels.

## Data Availability

Not applicable.
